# Blue blocking glasses worn at night in first year higher education students with sleep complaints: a feasibility study

**DOI:** 10.1186/s40814-018-0360-y

**Published:** 2018-11-01

**Authors:** Guillermo Perez Algorta, Anna Van Meter, Bernadka Dubicka, Steven Jones, Eric Youngstrom, Fiona Lobban

**Affiliations:** 1 Spectrum Centre for Mental Health Research, Division of Health Research, Lancaster University, Furness Building C73, Lancaster, LA14YT UK; 2 Yeshiva University, New York, USA; 3 University of Manchester, Manchester, UK; 4 University of North Carolina at Chapel Hill, Chapel Hill, USA

**Keywords:** Blue blocking light, Sleep, Activity, Mood, Circadian, Feasibility

## Abstract

**Background:**

Late adolescence and early adulthood is a period of highest incidence for onset of mental health problems. Transition to college environment has been associated with many risk factors such as the initial disruption—and subsequent irregularity—of the student’s sleep and activity schedule. We tested the feasibility of using blue blocking glasses (BBG) at night in first year higher education students with sleep complaints, to obtain preliminary evidence for the impact of BBG on sleep, activity, and mood.

**Methods:**

Participants were 13 first year undergraduates (from 10 different academic courses) living on campus for the first time with sleep complaints/disorders confirmed at screening via the Duke Structured Interview Schedule for Sleep Disorders. We used a 2-week, balanced crossover design (BBG vs placebo glasses; participants were unaware which was the active intervention) with computer-generated random allocation. Exploratory analyses provided descriptive and frequency summaries to evaluate feasibility of the intervention.

**Results:**

Preliminary evidence supports the feasibility and acceptability of the trial; almost all screened participants consented and completed the protocol with high adherence; missing data were negligible. Additionally, the effectiveness of BBGs to enhance sleep, mood, and activity levels in young adults was supported.

**Conclusions:**

The results of this feasibility trial suggest that BBG have potential as an inexpensive and feasible intervention for reducing sleep and circadian dysregulation in young adult students. A larger trial, following this successfully implemented protocol, is necessary to fully test the efficacy of BBG.

Late adolescence and early adulthood, a time associated with newfound independence and excitement for the future, is also the period of highest incidence for onset of mental health problems [1]. Research on the prevalence of mental health disorders on college campuses suggest that nearly half of students are affected [2, 3].The college environment is associated with many risk factors for vulnerable individuals, including easy access to alcohol and drugs, and reduced contact with family and existing support networks. Among the most powerful risk factors may be the initial disruption—and subsequent irregularity—of the student’s sleep and activity schedule [4]. In secondary school, most adolescents live structured lives, bound by obligations of school, extracurricular activities, family, parental control, and social life; but for many in college, classes are infrequent, and at least early on, there are fewer other obligations to meet, which can lead to irregular sleep and activity schedules [5, 6]. These risk factors may be particularly likely to increase risk for mood pathology; research has shown that depression is associated with insomnia and social isolation [7, 8]; and sleep disturbances and irregular social behavior are associated with mania and bipolar disorder [6, 9]. Research suggests that a young adult, deprived of only one night’s sleep, will experience greater levels of anger, anxiety, and stress than if s/he did sleep [7, 10]. Additionally, even relatively minor sleep disruptions due to social events or schoolwork can negatively impact daytime functioning and temporarily lower mood and decrease academic performance [11].

Disturbances of sleep and daily activity interfere with the circadian rhythm—the body’s clock [5], which is responsible for maintaining myriad biological processes, including sleep, metabolism, and energy [12]. The impact of dysregulated circadian rhythm is evident in other systems; for example, poor sleep is associated with weight gain [13]. Importantly, disruptions of the circadian rhythm and the resulting impact on mental and physical health are not due only to schedule disruptions; exposure to blue light—the type emitted from the sky (and from our omnipresent electronic devices)—is also a factor [12, 14]. Specifically, specialized retinal ganglion cells containing melanopsin (OPN4) track levels of blue and blue-green light (peak sensitivity ~ 470 nm) and signal the master clock in the brain (the suprachiasmatic nucleus of the hypothalamus [15]), to regulate other body processes to achieve the appropriate state of alertness (or sleepiness) based on environmental cues [12, 16]. In addition to time spent in front of the computer for academic reasons, young people tend to spend a significant amount of time in the evening engaging with technology—TV, video games, text messaging, etc. On average, they engage in more than four such activities after 9 pm [17]. This affects sleep significantly: Most adolescents and young adults get less than 8 h of sleep during the week—in many cases directly attributable to electronic device use [17], the consequences of which include depression, obesity, and poor academic performance [18].

These negative outcomes can derail an individual’s future [19]. Preventing depression, obesity, and poor academic performance during this period would be ideal, and evidence suggests that targeting students’ sleep problems can have broad, positive effects [20]. A new intervention, “virtual darkness” [14, 21], generated by wearing blue blocking glasses (BBG) may address this need. BBG work by “tricking” the body’s clock into believing that it is nighttime regardless of the blue light (whether from devices or the sky) in the environment [21]. Previous research has demonstrated that BBG can have a potent mood-stabilizing effect on inpatient adults with bipolar disorder [22] and can regulate sleep and improve mood in both healthy people [23] and postpartum women [24]. Importantly, BBG are a safe and well-tolerated intervention, positioning “virtual darkness” as a candidate preventive intervention that deserves further evaluation, particularly in groups in which sleep disturbance is prominent, like college students.

Our primary aims were to test the feasibility and acceptability of a balanced crossover design and to evaluate the effects of BBG in first year higher education students with sleep complaints. Changing sleep behaviors is often difficult [25], and given the unique living arrangements and social pressures on campus (i.e., students live together which creates an environment that might be incompatible with wearing glasses and/or with modifying one’s sleep, even if the BBG increased sleep drive, as expected), we determined that it was necessary to first test whether participants would follow the study protocol before launching a larger efficacy trial. Based on previous research [21, 22], we hypothesized that we would be able to successfully recruit our target sample (> 80% of *N* = 15) and that at least 70% of consented participants would complete the trial and demonstrate adequate adherence to the BBG protocol. Further, when BBG were worn at night for 3 h before target bedtime, we expected that preliminary evidence would show an effect in the expected direction of BBG improving sleep, activity, and mood, compared to a non-BBG intervention.

## Method

### Design

This study used a 2-week, balanced crossover design with computer-generated random allocation. The Faculty of Health and Medicine Research Ethics Committee (FHMREC), Lancaster University, approved the research protocol. Participants provided written informed consent. After protocol completion, participants were debriefed and received an Amazon voucher worth £75.

### Participants

We recruited 13 Lancaster undergraduates (from 10 different academic courses) from October 2015 to April 2016 via advertisements around campus. Sample size determination was based on pragmatic factors such as limited resources from pilot grant that payed for up to 15 participants. Inclusion criteria were first year undergraduates students living on campus for first time with sleep complaints/disorders confirmed at screening via the Duke Structured Interview Schedule for Sleep Disorders (DSISD; [26]). We excluded participants if they were unable or unwilling to comply with protocol; reported having severe retinal or corneal damage on both eyes; reported daily use of non-steroidal anti-inflammatory drugs, beta blockers, calcium-antagonist, or central stimulants like methylphenidate or venlafaxine; reported traveling outside the UK time zone during the past 2 months; reported changes in hormonal contraceptives during the past 2 months; or had brain dysfunction as observed during the screening interview.

### Measures

Participant characteristics (e.g., demographics) and questionnaires were collected online via Qualtrics software (2005), Version 3.5.0, Copyright © [2017].

Feasibility outcomes, such as protocol acceptance, were measured through recording of daily activities via Qualtrics (e.g., sleep diary completion and BBG wearing times) and corroborated by objective measurement of wear times of the actigraph. We collected data about recruitment timeline, and attrition and retention through database records. At the end of the study, the PI had a brief debriefing interview with participants, to ask about the experience of wearing BBG and whether participants accessed information on BBG from external sources during trial (which could create an expectancy effect confounding self-report of some measures of interest in future studies), and to get any suggestions about improving tolerability.

#### Mood ratings

The 7 Up-7 Down [27] is a 14-item self-report scale carved from the 73-item General Behavior Inventory [28]. It shows good internal consistency, high correlations with the full length scales, and good discriminative validity separating cases with mood disorders from other clinical complaints.

The Positive Affect–Negative Affect Schedule (PANAS) [29] is one of the most widely used rating scales to measure positive and negative emotions. It has 10 items of each valence rated on a Likert-type scale.

#### Chronotype

At screening, we used the Morningness/eveningness Questionnaire (MEQ) [30, 31].

### Procedure

The PI screened and consented all participants. At screening, participants were evaluated with the Duke Structured Interview Schedule for Sleep Disorders (DSISD; [26]). Baseline measures were repeated at days 3, 7, 10, and 14. Active (sleep log diary) and passive (Actigraphy, Actiwatch 2, Philips Respironics) activity and sleep data were collected daily, and sleep time and architecture based on peripheral arterial tone plus pulse oximetry (WatchPAT200, Itamar Medical [32] were collected at days 7 and 14.

Baseline data (days 1–3) was collected, and glasses and procedure were introduced to participants by PI. On day 4, participants previously matched by gender and morningness/evening preference at screening were randomly allocated (by computer) to wear either amber (active BBG; Uvex S1933X with required wave-length-blocking properties) or blue glasses (non-BBG; Uvex S1932X). Participants were told by PI that we were testing two pairs of glasses, each of which filtered different wavelengths of light, to reduce the likelihood of participant expectations about the effects of the BBG versus the blue glasses). Participants were instructed to put on the glasses at least 3 h before their target time to fall asleep until sleep onset each night (e.g., a participant with the goal of falling asleep at 1 am on day 4 would use the lenses after 10 pm with instructions to avoid taking them off until bedroom lights were off). A washout period occurred during days 8, 9, and 10 (no glasses). On day 11, participants crossed over to the other color of glasses. We asked participants to maintain their regular sleep-wake schedule during the 2-week period.

### Statistical analysis

Exploratory analyses provided descriptive and frequency summaries to evaluate feasibility of the intervention. Non-parametric tests (independent-samples Mann-Whitney *U* tests) compared medians between groups at night 7 and 14. Functional linear modeling of actigraphy data [33] investigated patterns of activity during intervention. Hierarchical linear modeling inspected preliminary evidence of impact of the intervention on self-report mood measures. Analyses were conducted in SPSS version 23 using the Actigraphy package [33] in *R* [34].

## Results

We report data for 12 participants (67% females; age mean 18.5, SD = .52; 5 White British, 4 Arabic, 2 Hispanic, and 1 Indian) who completed the 2-week protocol. One participant was unable to participate due to flooding on campus during scheduled data collection, and one participant was rejected for having traveled outside the UK time zone during the past 2 months.

All participants had sleep complaints, and 75% (*n* = 9) met criteria for at least one sleep disorder diagnosis (median age of onset ~ 15). Primary insomnia was most common (*n* = 6), followed by inadequate sleep hygiene (*n* = 4) (Table 1). None of the participants were currently taking medication for sleep problems. Three participants reported previously seeking mental health services; none currently were in pharmacological or/and psychotherapeutic treatment. The mean number of past mental health symptoms endorsed at baseline was 2.75 (SD = 3.31), and an average of 2.25 (SD = 3.31) symptoms were endorsed as current (range 0 to 12 symptoms, from a maximum possible of 36 symptoms evaluated using the mental health problems section of the DSISD [26]. Two participants disclosed past headaches. No alcohol or drug problems were reported, and three were considered heavy consumers of caffeine (one in the past) based on data collected via DSISD. Three of five females had irregular cycles; only one was using contraceptives before study.

**Table 1:** Sleep disorder diagnoses based on Duke structured interview schedule for sleep disorders by participant (*N* = 12)

Sleep Disorder Diagnoses and ID	a	b	c	d	e	f	g	h	i	j	k	l
Primary insomnia, current	X	X	X	X	X	X						
Circadian rhythm disorders	X											
Dyssomnia (Restless legs syndrome, poss.)	X											
Inadequate sleep hygiene	X	X	X	X								
Short sleeper		X										
Insomnia related with MH (depr, past)			X									
Breathing related sleep disorder, poss.				X								
Insomnia related with MH (anx, past)					X							
Parasomnia nos (Legs cramps, current)						X						
Minimal criteria for insomnia							X					
Parasomnia nos (Sleep paralysis, past)							X					
Insomnia related with MH (past&current)								X				
Nightmare disorder								X				
Primary Insomnia, past									X			
No diagnosis										X	X	X

Note: Letters in top horizontal axis represent each study participant. *X* meet diagnostic criteria for listed condition, *poss* possible, *depr* depression, *anx* anxiety

### Feasibility outcomes

All participants who were screened and eligible for the trial agreed to take part in the study. Only one participant refused to participate before screening because of level of commitment needed to complete daily assessments. We had a retention rate over the 2-week protocol of 92%; all participants completed the trial. We did not make any adjustments to the eligibility criteria or study design/expectations once recruitment began.

Additionally, adherence to the study requirements was high. The median number of days wearing glasses was 8 for BBG (range 5 to 8, expected number of days was 8), and 7.5 for non-BBG (range 4 to 9). The median number of minutes wearing BBG glasses per day was ~ 196 min (range 140 to 228, expected minutes 180), and ~ 205 min for non-BBG (range 138 to 212). No adverse events or unintended effects were reported.

All 12 participants wore the actigraph watch according to protocol instructions, and completed all daily assessments via Qualtrics. The level of missing data at item level was negligible. Seventy percent (*n* = 9) completed both peripheral arterial tone and pulse oximetry assessments at night 7 and 14. None of the participants reported having accessed information about BBG from external sources such as internet during study. These findings support the feasibility and acceptability of BBG as a sleep intervention in university students.

### Preliminary sleep, activity, and mood outcomes data

In terms of chronotype (morningness or eveningness preferences), the sample mean score was 14.58, SD = 3.48, range 9 to 20 from possible range of 0 to 24, where 0 means morningness preference.

In terms of pulse oximetry assessments, non-statistically significant differences between groups (BBG vs. non-BBG) were observed when comparing medians at night 7 and 14, *p*s *>* .05 (Table 2). However, those wearing amber glasses first tended to sleep longer and have fewer awakenings at night 7 than those wearing blue lenses, a favorable pattern that reversed at night 14 after participants switched to wearing blue lenses.

**Table 2:** Peripheral arterial tone plus pulse oximetry (WP-200) results at night 7 and 14 between groups

	Night 7		Night 14	
	Median	Range	Median	Range
Sleep time				
Amber first	7:08	5:20–8:18	6:24	4:24–7:32
Blue first	6:47	5:56–7:33	6:44	5:28–8:07
Sleep efficiency				
Amber first	85	83–95	84	62–86
Blue first	87	78–94	83	76–92
Awaking times				
Amber first	7	2–10	11	6–14
Blue first	9.5	4–15	8	4–16

Non-statistical differences between groups at night 7 and 14 were observed, *p*s > .06

**Fig. 1:**
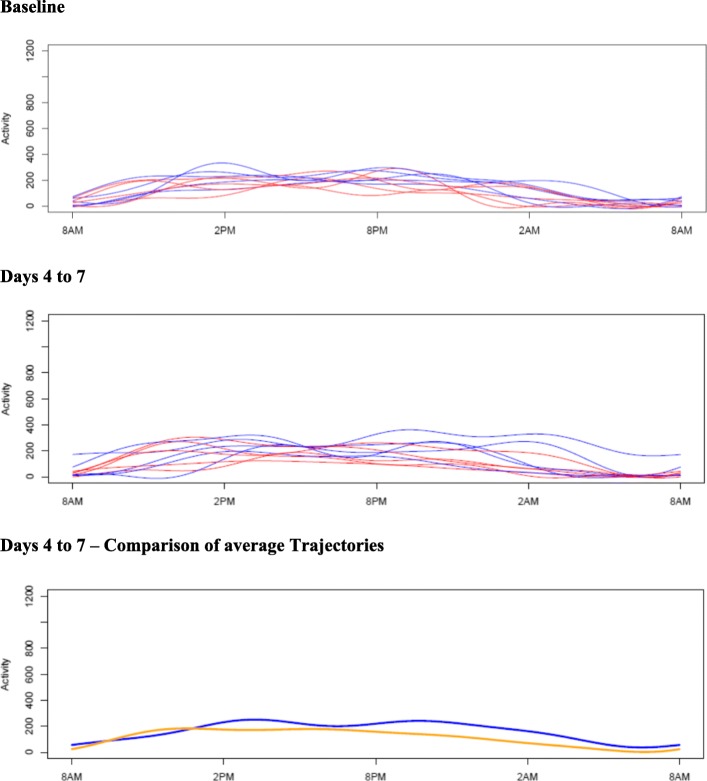
Aggregated 24 h activity level during baseline days versus activity level during days 4 to 7 and comparison of average activity trajectories

Graphical inspection of 24 h activity levels showed greater irregularity in those wearing blue, rather than amber glasses, during baseline days versus during days 4 to 7 (when participants received the intervention for first time) (Fig. 1). Comparing average trajectories found a lower level of activity in those wearing amber glasses during 8 pm to 2 am versus those wearing blue glasses. Group comparisons during days 11 to 14 were not possible because of battery performance problems with some devices during last days of intervention.

**Fig. 2:**
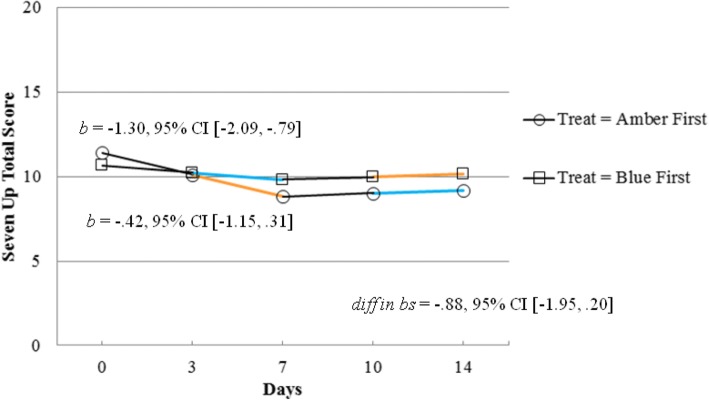
Manic-type symptoms trajectories (measured via Seven Up subscale) by treatment condition. Note. Colors orange and blue represent types of glasses. Black segments represent baseline or wash-up periods

Finally, in terms of mood change, we observed a decrease in hypomanic-type symptoms in those wearing amber glasses until day 7 relative to baseline (Fig. 2), differences were not observed during wash-out and when groups were allocated to blue glasses. Preliminary analyses using inferential statistics supported this observation (*b* = − 1.30, 95% CI [− 2.09, −.79]), and indicated a trend when analyzing the interaction between time × treatment-arm until day 7 (*diff in b*s = −.88, *p* < .10). No differences were observed on changes on depression scores, or on positive and negative affect scales.

## Comments

Recent studies have shown promising results in adults using blue-blocking glasses at night to improve sleep and mood. Our goal was to develop and test a protocol for use with sleep disturbed young adults, supplementing self-report measures with actigraphy while testing the feasibility and acceptibility of the intervention in a randomized crossover design. Participants’ report of number of days wearing BBG glasses at night and self-reported time wearing glasses both documented a high degree of acceptability for the intervention with young adults with sleep complaints. Of importance, no adverse effects were reported. The success in recruitment and retention, and the high level of task completion provided another indicator of adherence, with the low percentage of missing data showing the feasibility of this research project with this group of young adults with sleep complaints. This feasibility study, the first one using BBG with young adult students with sleep complaints living on campus, supports the feasibility of running this crossover design study. It also offers preliminary evidence about the potential use of this intervention to address challenges associated with risk factors of mental health onset or exacerbation of current symptoms for vulnerable individuals. In particular, the sleep profile observed in this sample resembles difficulties observed in clinical populations. The intervention was well accepted by this young group.

Preliminary evidence supported the positive impact of wearing BBG on sleep. Inspection of activity levels during days, and reduction of hypomanic-type symptoms in this sample, also provide preliminary evidence supporting the so called “deactivation hypothesis” associated with the impact of wearing BBG at night on noradrenergic pathways proposed by Henrisksen et al. [22].

In future studies with big samples of university students, there are some aspects that require further consideration, for example, wearing glasses maybe uncomfortable for some people, in particular for those already wearing regular glasses. Also, the potential impact of widespread publicity about blue light and BBG potentially may contribute to an expectancy effects (although these are less likely to affect actigraphy than self-report measures).

Because tinted glasses are widely used for fashion and other reasons, BBG could also offer a non-stigmatizing treatment option for this young adult population. It is also likely that BBG offer benefits beyond other options for reducing blue light exposure, such as apps that change the color composition of the images displayed on computers and hand-held electronics in the evening hours, because they block blue light from all environmental sources, including the television or LED lights. However, an important step in this research is to compare the relative impact of BBG and alternative methods directly. Finally, BBG are an appealing intervention in terms of adherence, compatible with student daily routines and recreational activities (e.g., use of social media and gaming via electronic devices).

## Limitations

This study needs replication given the small sample size, and potential impact of design characteristics, in particular length of wash-out period on carry-over effects of BBG. Notably, all findings observed ran in the expected direction, supporting the potential use of BBG to target difficulties associated with circadian dysregulation, such as sleep or mood difficulties. However, given the small sample size, our analyses were not adequately powered and should be considered preliminary, pending replication in a larger, adequately powered efficacy study.

## Conclusions

Wearing BBG at night shows promise as an inexpensive and feasible intervention for reducing problems associated with circadian dysregulation for young adults as well as adults. Larger studies with non-clinical and young clinical samples (inpatients and outpatients) should unpack the effects on cognitive functioning as well as mood and activity, as well as inform the development of more refined protocols for timing and otherwise improving sleep hygiene and implementation.

## References

[1] Kessler R (2005). Lifetime Prevalence and Age-of-Onset Distributions of DSM-IV Disorders in the National Comorbidity Survey Replication. Arch Gen Psychiatry.

[2] Blanco C (2008). Mental health of college students and their non-college-attending peers: results from the national epidemiologic study on alcohol and related conditions. Arch Gen Psychiatry.

[3] Norwalk K, Norvilitis JM, MacLean MG (2009). ADHD symptomatology and its relationship to factors associated with college adjustment. J Atten Disord.

[4] Carney CE (2006). Daily activities and sleep quality in college students. Chronobiol Int.

[5] Grandin L, Alloy L, Abramson L (2006). The social zeitgeber theory, circadian rhythms, and mood disorders: review and evaluation. Clin Psychol Rev.

[6] Shen G (2008). Social rhythm regularity and the onset of affective episodes in bipolar spectrum individuals. Bipolar Disord.

[7] Lund HG (2010). Sleep patterns and predictors of disturbed sleep in a large population of college students. J Adolesc Health.

[8] Stice E (2011). Relation of depression to perceived social support: results from a randomized adolescent depression prevention trial. Behav Res Ther.

[9] Gruber J (2011). Sleep matters: sleep functioning and course of illness in bipolar disorder. J Affect Disord.

[10] Minkel JD (2012). Sleep deprivation and stressors: evidence for elevated negative affect in response to mild stressors when sleep deprived. Emotion.

[11] Short MA (2013). The impact of sleep on adolescent depressed mood, alertness and academic performance. J Adolesc.

[12] Zelinski EL, Deibel SH, McDonald RJ (2014). The trouble with circadian clock dysfunction: multiple deleterious effects on the brain and body. Neurosci Biobehav Rev.

[13] Chaput J-P (2008). The association between sleep duration and weight gain in adults: a 6-year prospective study from the Quebec family study. Sleep.

[14] Phelps J (2008). Dark therapy for bipolar disorder using amber lenses for blue light blockade. Med Hypotheses.

[15] Foster RG (2005). Neurobiology: bright blue times. Nature.

[16] Vandewalle G (2007). Brain responses to violet, blue, and green monochromatic light exposures in humans: prominent role of blue light and the brainstem. PLoS One.

[17] Calamaro CJ, Mason TBA, Ratcliffe SJ (2009). Adolescents living the 24/7 lifestyle: effects of caffeine and technology on sleep duration and daytime functioning. Pediatrics.

[18] Owens J (2014). Insufficient sleep in adolescents and young adults: an update on causes and consequences. Pediatrics.

[19] Evans NJ, et al. Student development in college: Theory, research, and practice. San Francisco: Wiley; 2009.

[20] Freeman D (2017). The effects of improving sleep on mental health (OASIS): a randomised controlled trial with mediation analysis. Lancet Psychiatry.

[21] Burkhart K, Phelps JR (2009). Amber lenses to block blue light and improve sleep: a randomized trial. Chronobiol Int.

[22] Henriksen TE (2016). Blue-blocking glasses as additive treatment for mania: a randomized placebo-controlled trial. Bipolar Disord.

[23] Sasseville A (2006). Blue blocker glasses impede the capacity of bright light to suppress melatonin production. J Pineal Res.

[24] Bennett S (2009). Use of modified spectacles and light bulbs to block blue light at night may prevent postpartum depression. Med Hypotheses.

[25] Irish LA (2015). The role of sleep hygiene in promoting public health: a review of empirical evidence. Sleep Med Rev.

[26] Edinger J (2009). Reliability and validity of insomnia diagnoses derived from the Duke structured interview for sleep disorders. Sleep.

[27] Youngstrom EA (2013). The 7 Up 7 Down Inventory: a 14-item measure of manic and depressive tendencies carved from the General Behavior Inventory. Psychol Assess.

[28] Depue RA (1981). A behavioral paradigm for identifying persons at risk for bipolar depressive disorder: a conceptual framework and five validation studies. J Abnorm Psychol.

[29] Watson D, Clark LA, Tellegen A (1988). Development and validation of brief measures of positive and negative affect: the PANAS scales. J Pers Soc Psychol.

[30] Horne JA, Östberg O. A self-assessment questionnaire to determine morningness-eveningness in human circadian rhythms. Int J Chronobiol. 1976;4:97–10. 1027738

[31] Košćec A, Radošević-Vidaček B, Kostović M (2001). Morningness–eveningness across two student generations: would two decades make a difference?. Personal Individ Differ.

[32] Hedner J (2011). Sleep staging based on autonomic signals: a multi-center validation study. J Clin Sleep Med.

[33] Wang J (2011). Measuring the impact of apnea and obesity on circadian activity patterns using functional linear modeling of actigraphy data. J Circadian Rhythms.

[34] R Core Team (2014). R: A language and environment for statistical computing.

